# Large-Scale Mapping and Predictive Modeling of Submerged Aquatic Vegetation in a Shallow Eutrophic Lake

**DOI:** 10.1100/tsw.2002.194

**Published:** 2002-04-09

**Authors:** Karl E. Havens, Matthew C. Harwell, Mark A. Brady, Bruce Sharfstein, Therese L. East, Andrew J. Rodusky, Daniel Anson, Ryan P. Maki

**Affiliations:** South Florida Water Management District, West Palm Beach, FL 33406, USA

**Keywords:** submerged vegetation, mapping, modeling, Chara, shallow lakes

## Abstract

A spatially intensive sampling program was developed for mapping the submerged aquatic vegetation (SAV) over an area of approximately 20,000 ha in a large, shallow lake in Florida, U.S. The sampling program integrates Geographic Information System (GIS) technology with traditional field sampling of SAV and has the capability of producing robust vegetation maps under a wide range of conditions, including high turbidity, variable depth (0 to 2 m), and variable sediment types. Based on sampling carried out in AugustœSeptember 2000, we measured 1,050 to 4,300 ha of vascular SAV species and approximately 14,000 ha of the macroalga Chara spp. The results were similar to those reported in the early 1990s, when the last large-scale SAV sampling occurred. Occurrence of Chara was strongly associated with peat sediments, and maximal depths of occurrence varied between sediment types (mud, sand, rock, and peat). A simple model of Chara occurrence, based only on water depth, had an accuracy of 55%. It predicted occurrence of Chara over large areas where the plant actually was not found. A model based on sediment type and depth had an accuracy of 75% and produced a spatial map very similar to that based on observations. While this approach needs to be validated with independent data in order to test its general utility, we believe it may have application elsewhere. The simple modeling approach could serve as a coarse-scale tool for evaluating effects of water level management on Chara populations.

### INTRODUCTION

Submerged aquatic vegetation (SAV) plays a keystone role in shallow lakes, providing habitat for fish, birds, and other wildlife, supporting epiphyton that can be an important source of carbon and energy in the lake food web, and directly affecting water quality. Plants stabilize sediments with their roots, reduce water flow velocity and shear stress on the sediment surface due to wave attenuation[1], and trap sediments among their roots. Along with their epiphyton, submerged plants sequester phosphorus from the sediments and water column[2,3,4]. In lakes with abundant and widespread SAV, there is a positive feedback system that can prevent phytoplankton from reaching high densities[5,6] and is a strong in-lake sink for phosphorus[7]. In contrast, lakes without SAV generally are turbid and phytoplankton-dominated[8,9], and their sediments are more prone to wind- and wave-driven sediment resuspension.

A solid understanding of SAV dynamics is key to successful management of shallow lakes[9]. As such, there has been considerable focus on SAV in shallow lake research, predictive modeling, and long-term ecosystem management. Research has been done to identify SAV responses to sediment type[10], water depth[11], transparency[12], bottom slope[13], and other attributes. Modeling has ranged from relatively simple empirical relationships[12,13] to complex mechanistic models of lake hydrodynamics and water quality[14,15]. Methods of long-term assessment are highly variable and include remote sensing[16,17,18], transect sampling[19], and point sampling[11,20].

In this study, we address some of the challenges associated with the assessment and modeling of SAV in large, shallow lakes and estuaries. These include (1) the large spatial extent, (2) the sometimes great spatial heterogeneity in plant abundance and environmental conditions, and (3) the inability to detect plants with remote sensing if water is turbid. We describe a method that was used to rapidly sample and map SAV over an area of approximately 20,000 hectares and that can be used in an unbiased manner under a wide range of conditions (varying depth, turbidity, sediment type). Simple decision-tree models also were developed to explain the observed spatial variation in SAV over this large landscape. The macroalga Chara was the focus of the decision-tree modeling because it presently dominates the biomass in the study lake[21] and in many other shallow eutrophic lakes, especially those undergoing recovery from cultural eutrophication[20,22,23]. The main objective of the modeling was to determine whether water depth, a standard output parameter from regional hydrologic model runs, could be used (possibly in concert with information on sediment type) to predict the spatial distribution of Chara during restoration planning.

### METHODS

#### Study Sites

The research was carried out on Lake Okeechobee, a large (area ~1,800 $km^2$), shallow (mean depth ~2.7 m) lake in Florida, U.S. ($27^o$ 00‘ N latitude, $80^o$ 50‘ W longitude). The lake has three distinct zones. A littoral zone of emergent, submerged, and floating leaf plants along the west and south shoreline encompasses ~400 $km^2$ of the lake. At low to moderate lake levels, it receives most of its water inputs from direct rainfall and is oligotrophic[24]. A central pelagic zone encompasses 1,200 $km^2$ of the lake and has nutrient-rich turbid water due to resuspension of mud bottom sediments by wind and waves. It does not support any vascular plants. A pelagicœlittoral interface (near-shore) zone is the most dynamic region of the lake. It encompasses ~200 $km^2$ and supports vascular plants and Chara under low water conditions (stage <3.9 m above sea level) but is phytoplankton dominated when water levels are high (stage >4.7 m)[21,25]. The present study focuses on the near-shore zone.

#### Sampling Methods and Map Development

In order to carry out a systematic program of SAV sampling, a coverage of the lake surface was overlaid onto a rectangular grid of 500 þ 500 m cells in ARC/INFO. This scale of resolution reflected a consensus view among the investigators regarding what was practical and what was adequate to capture most of the spatial variation observed in the field. A pre-existing coverage[26] of the littoral zone was laid onto the map, and the common cells were clipped from the final coverage, as was the deeper central pelagic region. This resulted in a near-shore grid of approximately 2,000 cells. Coordinates for the grid cell center points were loaded into a Trimble Pathfinder GPS unit (differentially corrected) for use in navigating to the sampling sites. A simple program was set up in the data logger so that users could enter information regarding water depth, Secchi depth, sediment type, presence vs. absence of SAV taxa, and a qualitative estimate of overall plant biomass (sparse, moderate, dense) based on the volume of material collected in a large plastic sorting tray.

Sampling started in late August and was over 90% complete by the end of September 2000. Sampling began near the lakeshore and proceeded lakewards along each row of cells until two consecutive cells were encountered with no SAV. At the end of sampling, data had been collected from 1,478 locations. The boat operator navigated to the site by GPS, where water depth was measured with a calibrated line and Secchi depth was measured with a 20-cm black-and-white disk. Plant sampling was done using a tool constructed of two standard garden rakes bolted together at midpoint to create a tonglike device. The degree of opening was constrained by placing a chain between the two handles, such that three replicate samplings with the device removed ~1 $m^2$ of bottom cover. When the rakes were in a closed position (collecting plants), the distance between adjacent tines was approximately 10 mm. Plants were placed into a plastic tray, sorted by species, and the information described above was entered into a data logger. Sediment type was determined by inspection of material brought up in the rakes or by observation from the boat where water transparency was good. Sampling was possible up to a maximal depth of approximately 2 m.

Field data were downloaded from the GPS logger into ARC/INFO, where maps were developed for each of the measured attributes and spatial extents were calculated in hectares. It was assumed that data collected at the center point of a grid cell represented that of the entire cell. While this may introduce error into the results, it was a necessary assumption given the large spatial scale of sampling.

### Model Development

A set of simple decision-tree models was constructed in an Excel spreadsheet in an attempt to explain the observed spatial distribution of Chara. Models were constructed based on (1) depth alone, and (2) sediment type and depth. For each model, a range of cut-off points (e.g., for sand, critical depth >0.5 m = no Chara, depth ≤0.5 m = Chara) were tested at 0.5-m intervals until the best fit between observed and predicted patterns of presence/absence were observed. Model development involved sorting the spreadsheet data by depth and/or sediment type, identifying the cutoff point(s), and then classifying each sampling point (each row in the spreadsheet) as —Chara present“ or —Chara absent“ based on whether or not its depth fell below or above the cutoff point for each sediment category. These model predictions then were compared with a column of data indicating actual presence and absence information to determine overall accuracy. Model results also were mapped in GIS and compared visually to the maps based on observed data.

Although information was available regarding Chara relative density at the various sites, we used only presence/absence, which was judged to be more robust in terms of unbiased sampling across all sediment types and water depths. Secchi data were not used in the model development process, although they were collected during the mapping effort. Secchi depth is a very good predictor of Chara occurrence in the lake[21], and this will be taken into consideration when developing a more complex hydrodynamic water quality model.

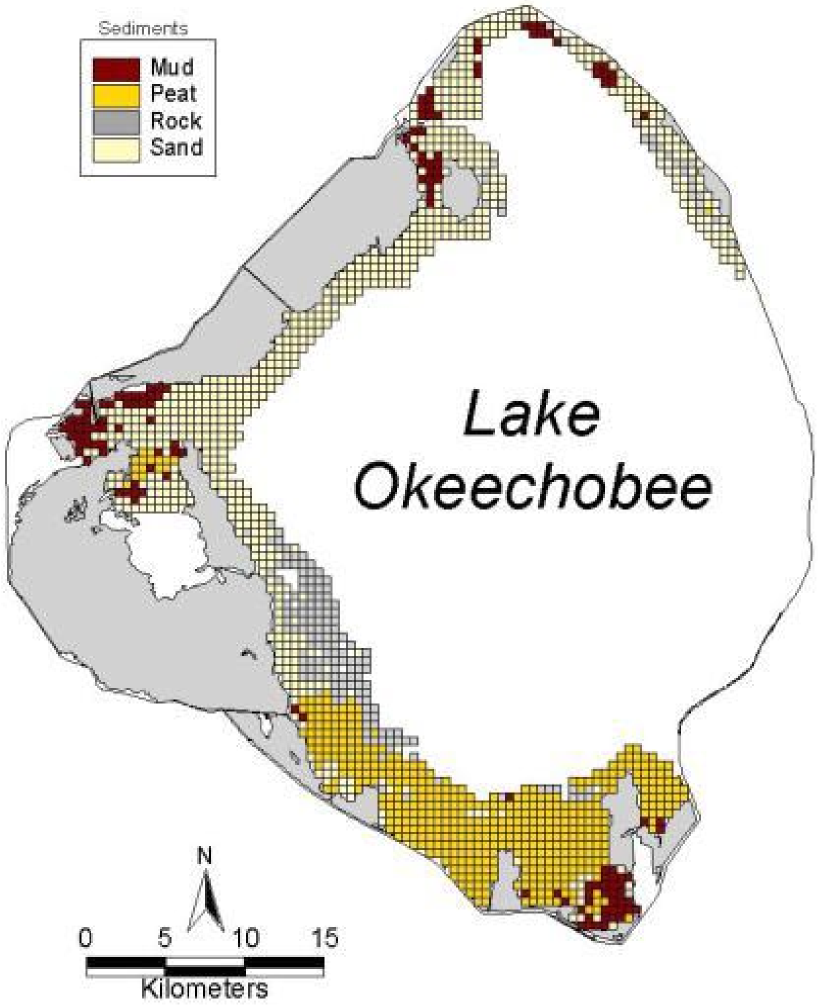

**FIGURE 1.** GIS maps showing (A) the distribution of mud, peat, rock, and sand sediments; (B) water column depths; and (C) Secchi depths, as a percent of water column depth; in the near-shore region of Lake Okeechobee where submerged aquatic vegetation occurred in late summer 2000. Each square is 500 x 500 m. The light gray shaded area along the western shoreline is the lake's littoral zone of emergent vegetation. The white area is deeper water that was not mapped.

### RESULTS

#### Mapping

In the near-shore region where submerged vegetation occurred, there were four dominant sediment types (Fig. 1A). Mud sediments comprised 10% (3,525 ha) of the area, peat comprised 33% (12,025 ha), rock 12% (4,300 ha), and sand 45% (16,125 ha) of the sampled region. Peat dominated in the south, while sand was the predominant sediment type in the northwest and north. Water depths in the sampling region (Fig. 1B) ranged from less than 10 cm to over 2 m. Shallowest locations were in the bays at the south and west regions. In total, depths were between 0œ25 cm in 7% (2,550 ha) of the sampling area, between 26œ50 cm in 12% (4,350 ha), 51œ75 cm in 18% (6,400 ha), 76œ100 cm in 13% (4,850 ha), and >100 cm in 50% (17,825 ha) of the area. Water depths at any given location did not vary substantially during the sampling program

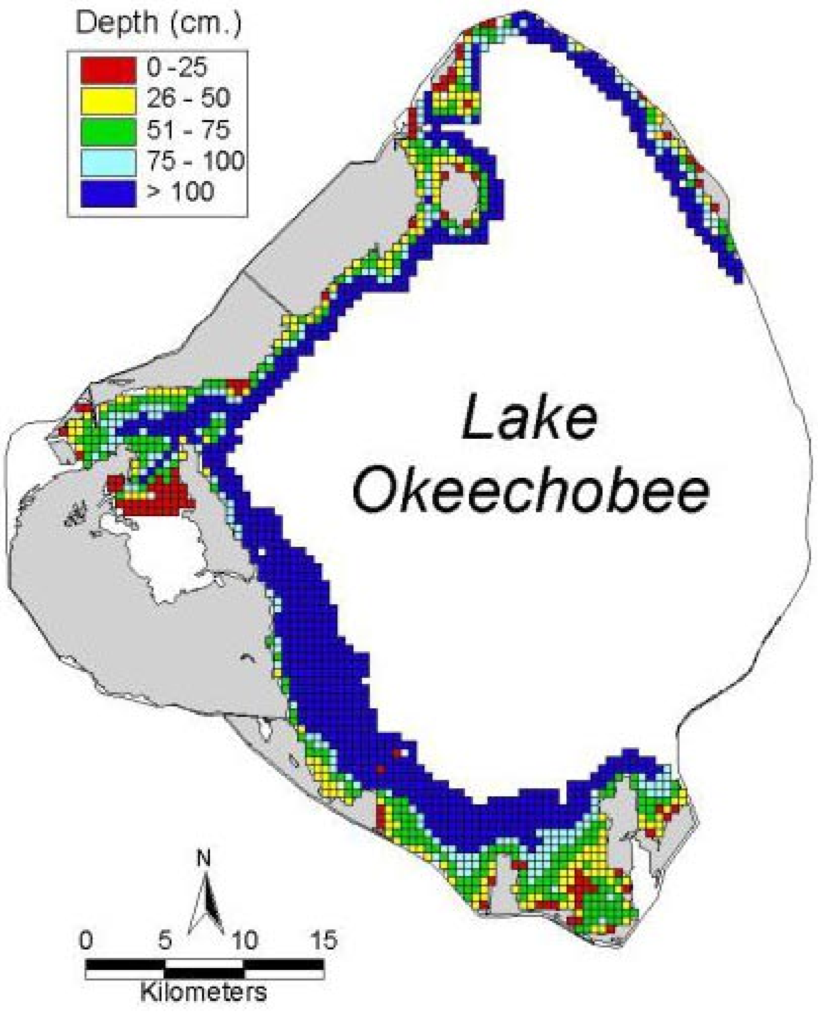

**FIGURE 1B.**

relative to this range of observations. Secchi transparencies (as a percent of total depth) ranged from less than 10 to 100%, with a large region of maximal transparency occurring in the south and southwest regions (Fig. 1C). In total, relative Secchi depths were between 0œ25% in 9% (3,225 ha) of the sampling area, between 26œ50% in 31% (11,250 ha), 51œ75% in 28% (10,000 ha), and 76œ 100% in 32% (11,400 ha) of the area.

Vallisneria americana occurred primarily as two large stands in the south and west bays and as a narrow fringe along the northwest shore (Fig. 2A). In total, there was an estimated 4,273 ha of V. americana. Hydrilla verticillata occurred almost exclusively in the south and west bays and had an estimated coverage of 2,649 ha (Fig. 2B). Potamogeton illinoensis occurred only in the south end of the lake, with an estimated coverage of 1,000 ha (Fig. 2C), while Ceratophyllum demersum occurred both in the south and west, with an estimated coverage of 1,049 ha (Fig. 2D). The most wide-spread coverage was documented for the macroalga Chara spp., which occurred primarily in the south and southwest, with a total of 13,945 ha (Fig. 2E). When all species are considered, there was a total of 17,750 ha of submerged vegetation in the lake at the end of the 2000 growing season.

The occurrence of Chara was strongly associated with peat sediments (Fig. 3A), water depths below

1.5 m (Fig. 3B), and high percent Secchi depth (Fig. 3C).

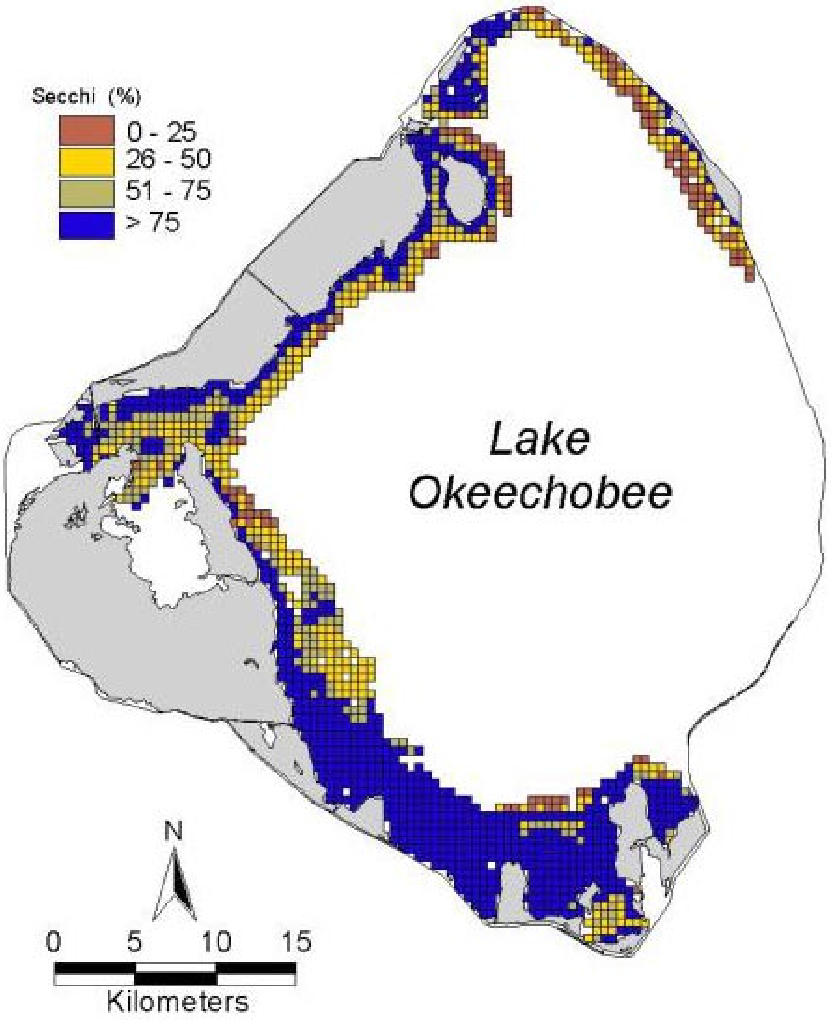

**FIGURE 1C**

### Modeling

The first decision model considered only water depth as an explanatory variable for Chara occurrence. The data set was screened at 0.5-m depth intervals until a breakpoint was identified that best accounted for the observed Chara distribution. A model with a decision based on the criterion [Chara present if depth $\leq$1.5 m, Chara absent if depth >1.5 m] gave the best fit but had a prediction accuracy of 55% (Fig. 4A). The model predicted widespread occurrence of Chara in locations along the south, west, and northwest shoreline where the plant was not actually found.

The second model incorporated sediment type (mud, peat or rock, sand) as a first variable and then calibrated within sediment types for depth as described above. The best resulting decision tree was as follows:

Mud ②Chara absent Peat or Rock ②Chara present if depth ≤1.5 m, Chara absent if depth >1.5 m
Sand ②Chara present if depth ≤0.5 m, Chara absent if depth >0.5 m

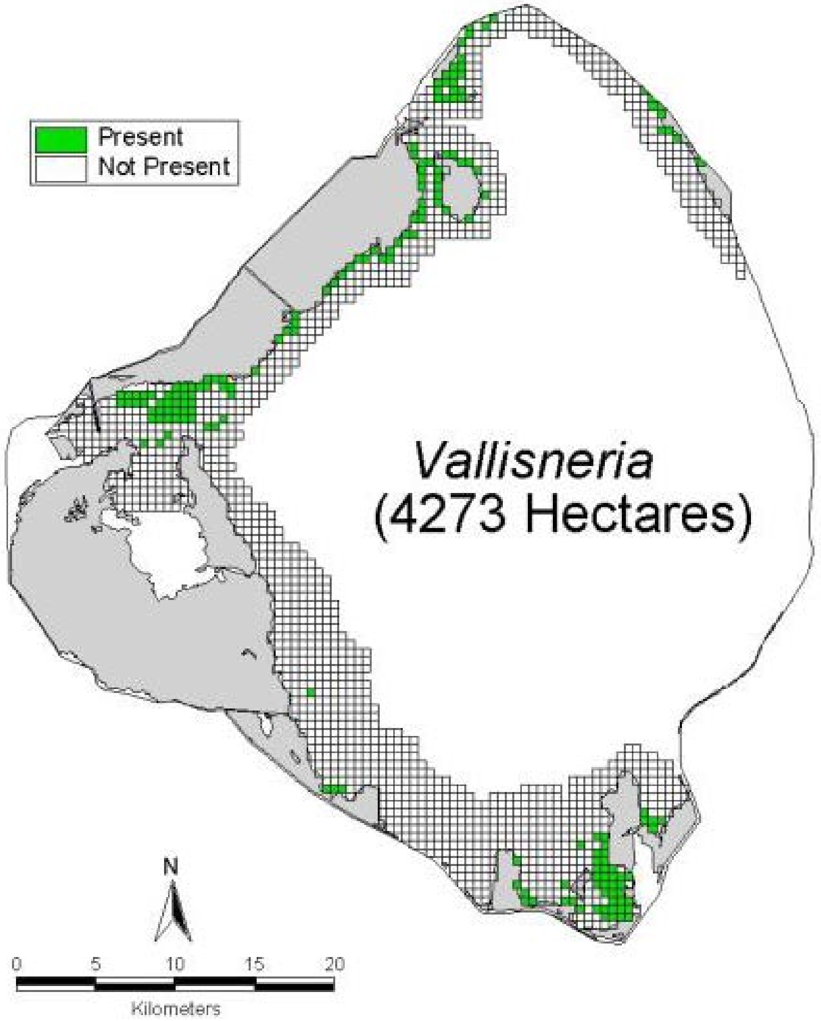

**FIGURE 2**. GIS maps showing the spatial distribution of (A) Vallisneria americana, (B) Hydrilla verticillata, (C) Potamogeton illinoensis, (D) Ceratophyllum demersum, and (E) Chara spp. in Lake Okeechobee during late summer 2000. Each square is 500 x 500 m. The light gray shaded area along the western shoreline is the lake's littoral zone of emergent vegetation. The white area is deeper water that was not mapped.

The model using these three decision rules correctly predicted 75% of the observed spatial distribution of Chara (Fig. 4B). The only notable area of underprediction occurred in the north and along the western shore, where qualitative sampling (into categories of sparse, moderate, dense) suggested that the biomass of Chara was sparse.

## DISCUSSION

### GIS-Based Sampling

In this study, a GIS-based sampling program was used to effectively collect SAV and environmental data in a large, turbid lake. A total of 1,478 sites were sampled in a period of just under 2 months. Data processing was facilitated by using GPS units with pull-down menus for quick entry of information on sediment, plant species, water depth, and Secchi transparency.

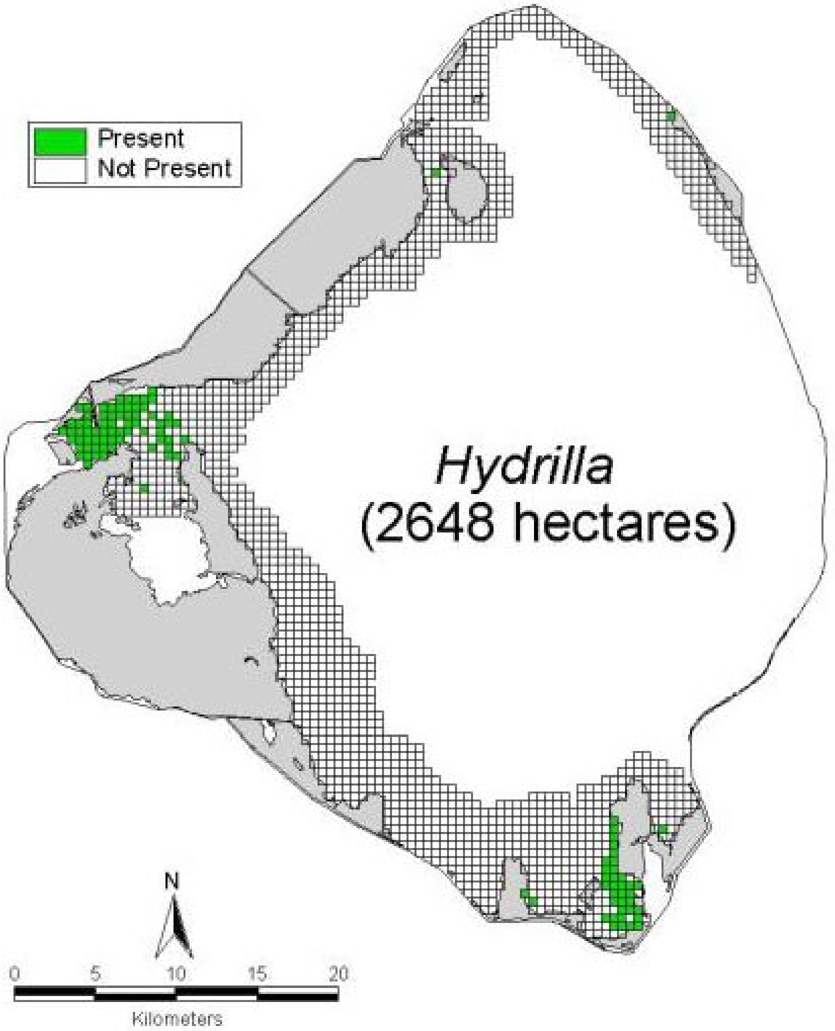

FIGURE 2B

Collected data were directly downloaded into GIS, and map development proceeded as the project moved forward. This had two benefits: (1) it allowed field crews to immediately see the results for their work, and (2) it helped in selection of sampling regions on successive days of the project.

Because plants were physically removed from the lake bottom and sorted on the boat to species, documentation of presence/absence is not affected by environmental conditions (in particular, turbidity and depth) that could interfere with more commonly used methods, such as aerial photography[27,28]. This ability to obtain consistent samples is of particular importance in Lake Okeechobee, where Secchi transparencies can vary from below 10 to over 150 cm, sometimes reaching the lake bottom[29].

Judging from our field experiences, the rake tongs method might not be as effective in collecting small plants or very sparse plants where sediments are particularly compacted (e.g., hard packed sand). Until a quantitative comparison has been made with other sampling methods in the lake, we restrict data analysis from the mapping project to the presence/absence information. Other methods are used to quantify biomass of SAV in Lake Okeechobee but at a coarser spatial intensity. For example, we determine biomass approximately bimonthly at sites arranged along 15 transects perpendicular to the lake shore[25]. Divers underwater harvest all plants enclosed by 0.5-$m^2$ quadrats (3 per site). Comparative analyses have shown that this approach may be more effective than raking (with a single rake) for documenting the occurrence of small and rare plants[30]. However, it remains to be seen how our quadrat sampling compares to sampling with a two-part rake tong that can effectively grasp and hold plant material. The rake tongs device (or similar oyster tongs) are used in other large-scale SAV mapping programs, such as that carried out on the Potomac River[31].

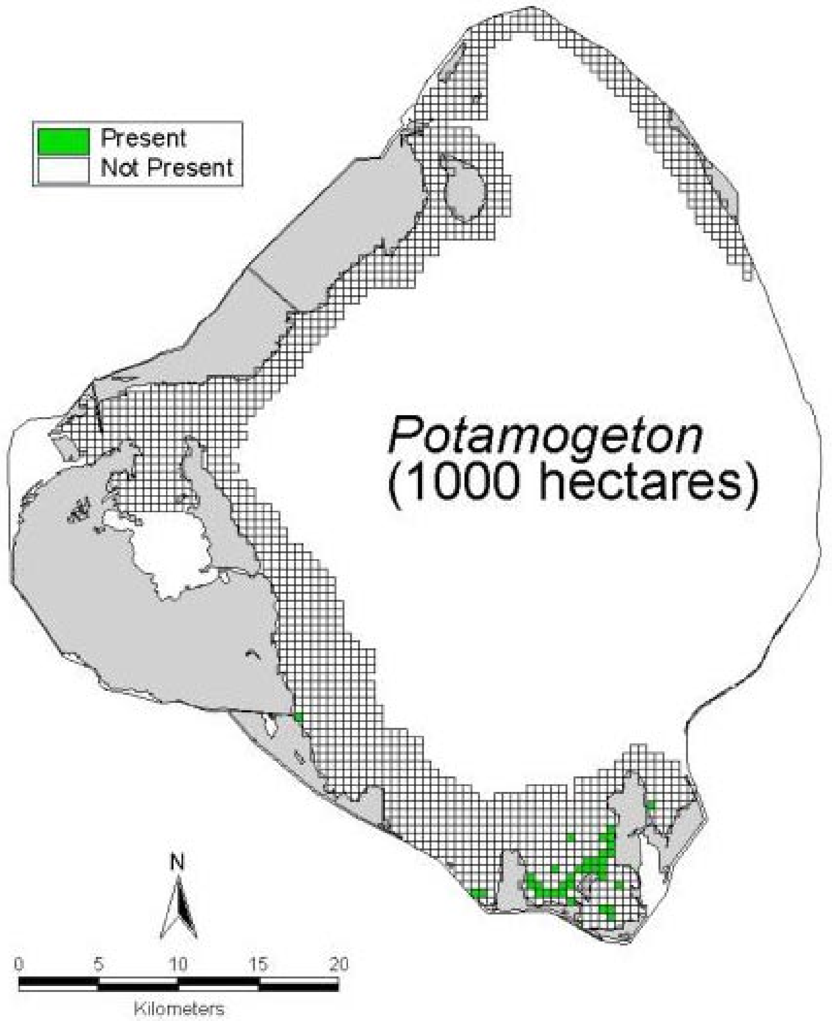

**FIGURE 2C**

### Historical Comparisons

Although this is the first study to actually —map“ the submerged vegetation in Lake Okeechobee, there are previous data collected from less spatially intensive transect sampling programs. In particular, Zimba et al.[15] sampled SAV along 60 transects perpendicular to the shoreline in summer 1989œ1991. They constructed crude spatial maps for the dominant species by connecting the outermost points along each transect where the species was found, and then determining the area enclosed between that outer boundary and the lake shore with GIS. In the 3 sampling years, summer average lake stages (surface elevation above sea level) were approximately 3.5, 3.3, and 3.8 m, respectively. This compares with a mean stage of approximately 3.6 m in the present study. Zimba et al.[15] estimated that there were 4,200√8,200 ha of V. americana (vs. 4,300 ha at present), 3,800√4,800 ha of H. verticillata (vs. 2,650 ha at present), 4,900√8,700 ha of P. illinoensis (vs. 1,000 ha at present), and 11,900√12,800 ha of Chara spp. (vs. 13,950 ha at present). Considering that a decade separates the two sampling events and that the methods were quite different, these results are surprisingly similar and indicate a degree of consistency in the lake‘s SAV community. The exception is P. illinoensis, which has displayed a substantial reduction in spatial extent.

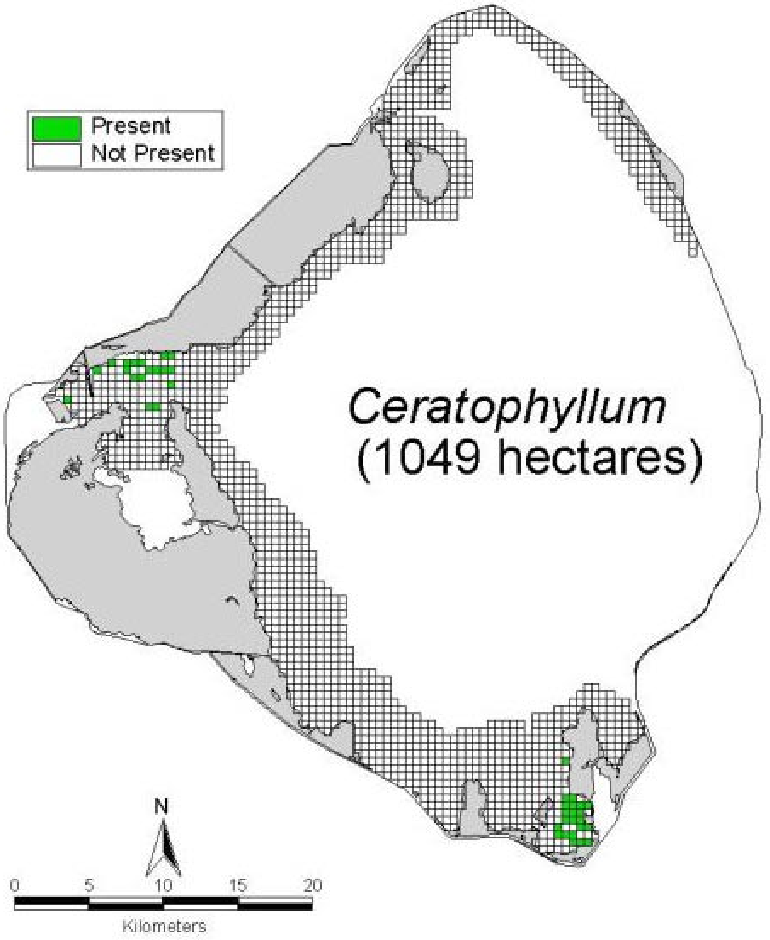

FIGURE 2D

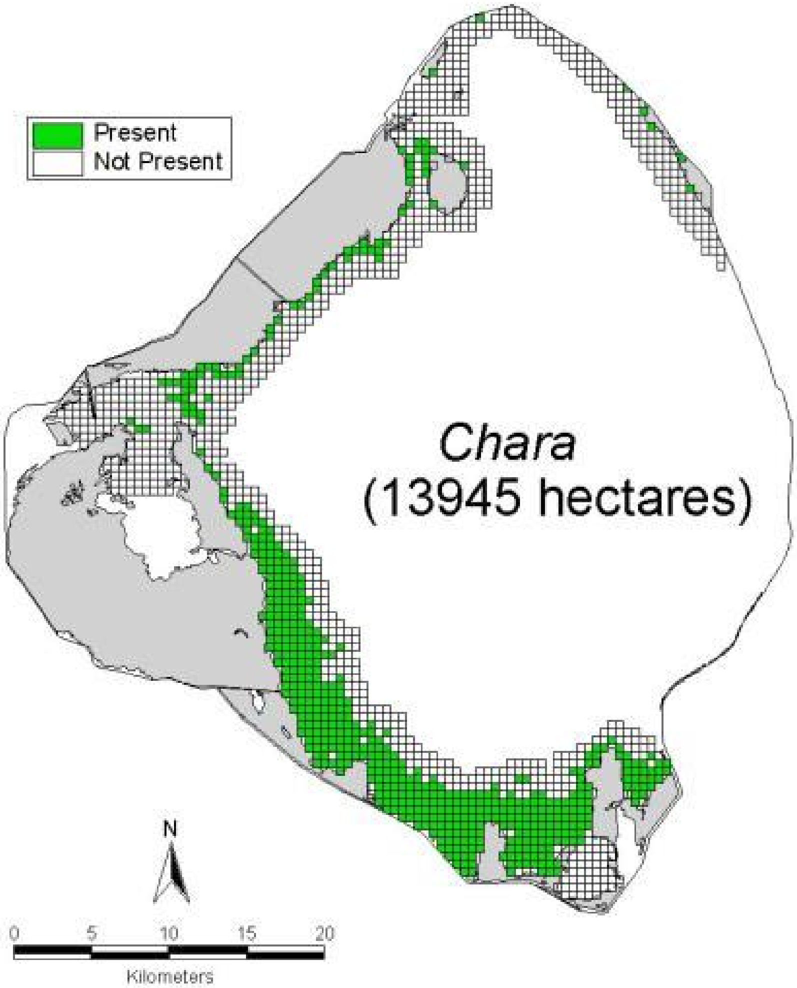

**FIGURE 2E**

We attribute this to (1) a period of prolonged high water levels in the late 1990s, and (2) impacts of a hurricane in fall 1999[25]. In contrast to *V. americana* and *Chara spp*., which seem to have widespread viable seeds and oospores, respectively, the sedimentary seed bank for P. illinoensis appears to have considerably depleted in this lake[32].

### Factors Controlling the SAV

In this study, vascular SAV was observed primarily in large southern and western beds where it was known to have occurred in previous years. As such, any predictive modeling of these plants must take into consideration the past history of occurrence as well as environmental conditions during the present growing season. Therefore the subsequent discussion is restricted to Chara spp., which is an annual plant in Lake Okeechobee, disappearing almost completely during winter months when colder and deeper conditions occur[33]. In this study, the occurrence of Chara spp. was strongly associated with peat sediments. Approximately 75% of sites with peat supported Chara, as compared to 10 to 30% of other sites. It is unknown whether there are physical or chemical characteristics of peat that are favorable for the growth of Chara, or whether it just .

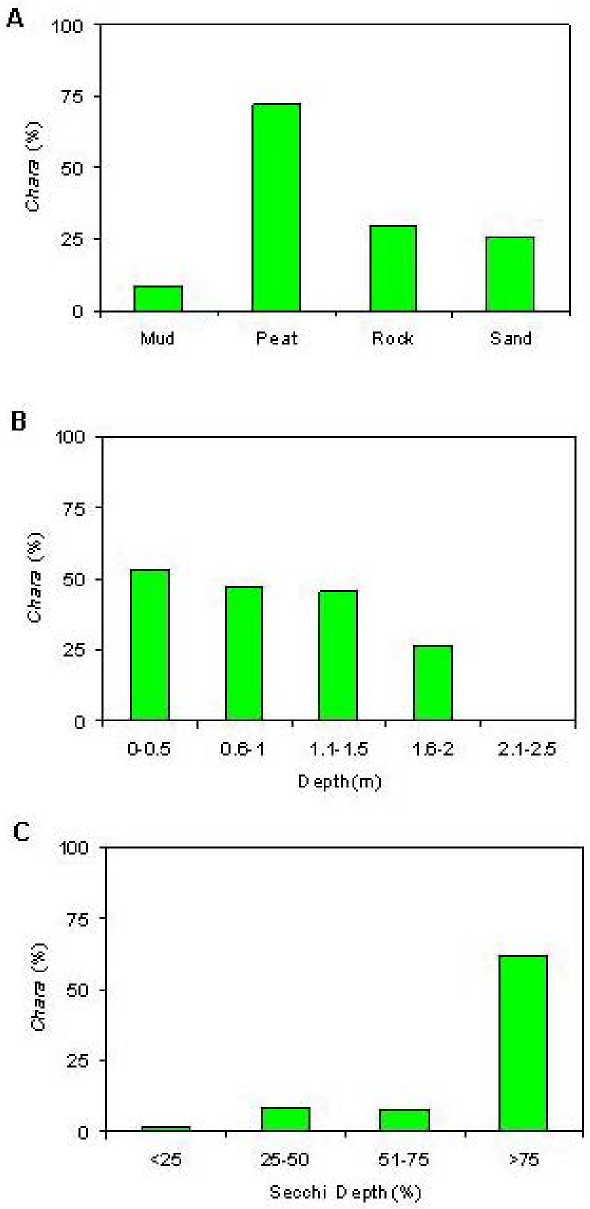

**FIGURE 3**. Relationship between occurrence of Chara spp. in the near-shore region of Lake Okeechobee in late summer 2000 and (A) sediment type; (B) water column depth; and (C) Secchi depth, as a percent of water column depth.

occurs coincidentally with peat in the southern lake region. Another explanation is that Chara is absent from certain locations (mud sediments) because those locations support vascular plants (Vallisneria and Hydrilla) that outcompete Chara. It is known that many Chara-dominated lakes reported in the literature have peat sediments[20,23]. A final explanation is that Chara may occur primarily at the south end of the lake because it reflects a remnant population from the Everglades, which once was contiguous with the lake.

Chara occurrence in Lake Okeechobee also was associated with depth of the water column. Sites with depths of 1.5 m or lower displayed approximately 50% occurrence of Chara, while this number dropped to 25 and 0% at depths in excess of 1.5 and 2.0 m, respectively. As has been documented previously[21], there also is a strong relationship between Chara occurrence and the ratio of Secchi to total depth. Where that ratio was greater than 75%, there was a high probability

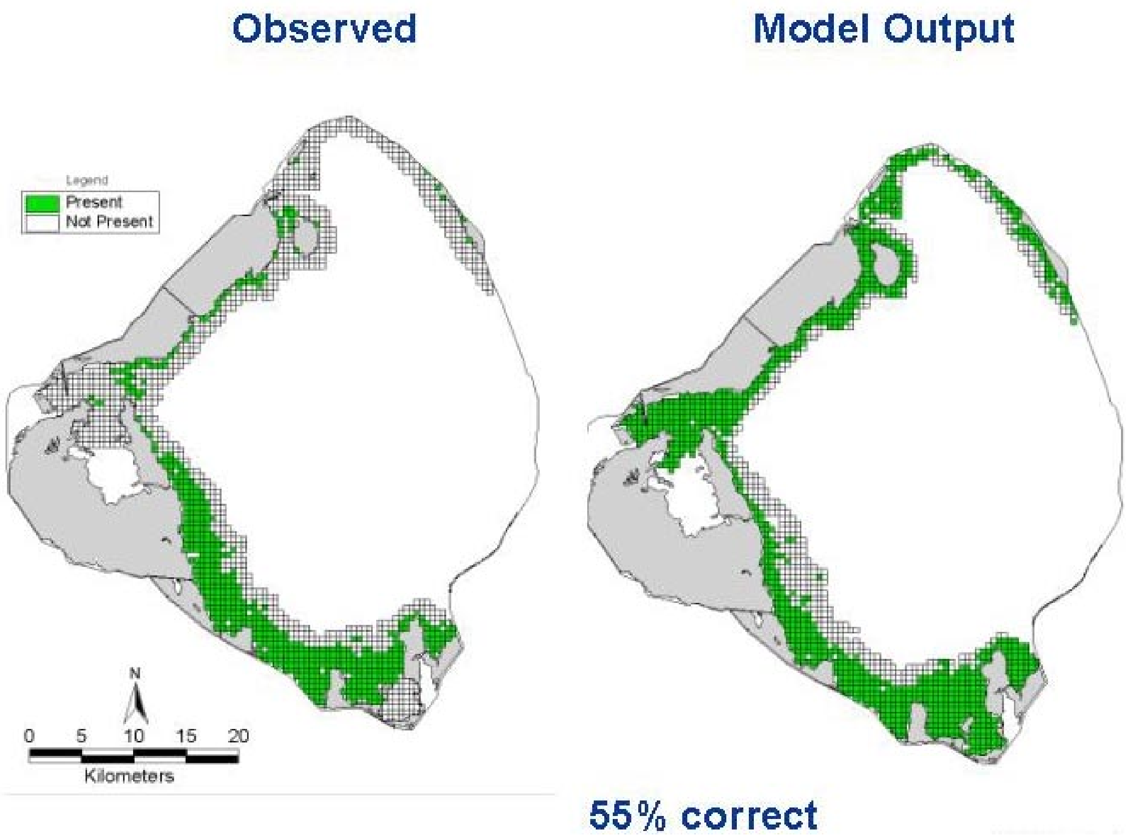

**FIGURE 4.** Comparison of results of GIS mapping of Chara spp., based on actual observations in late summer 2000 vs. the predictions from (A) a decision model using only water depth as a predictor, and (B) a model using sediment type and then water depth as stepwise predictors. The white area is deeper water that was not mapped.

of encountering Chara; where the ratio was lower than 75%, the probability of occurrence was low. Of the variables examined, this one had the most dramatic breakpoint, suggesting that light availability may be the critical attribute in mechanistic modeling of Chara. This reflects the findings of past research with this plant and the general approach that has been taken in Chara modeling in other ecosystems[14,15,34].

### Decision Models for Chara

One objective of this study was to determine if a simple model could be developed for predicting Chara responses to different lake management scenarios. The long-term goal of our lake modeling program is to develop an integrated hydrodynamic√water quality√SAV model, similar to that developed by Best et al.[34]. South Florida now is engaged in a multibillion-dollar project to restore the greater Everglades ecosystem, which includes Lake Okeechobee. Early screening of project components includes evaluations by scientists of potential system responses. For the lake, input data are comprised of daily hydrographs for a 30-year simulation period. Following a validation exercise in 2001œ2002, it should be possible to apply the sediment/depth decision-tree model for Chara to this management issue. The underlying concept is to provide a quantitative prediction about one system component (Chara) until such time that a more complex model can provide more detailed information about multiple components (Chara and vascular SAV species).

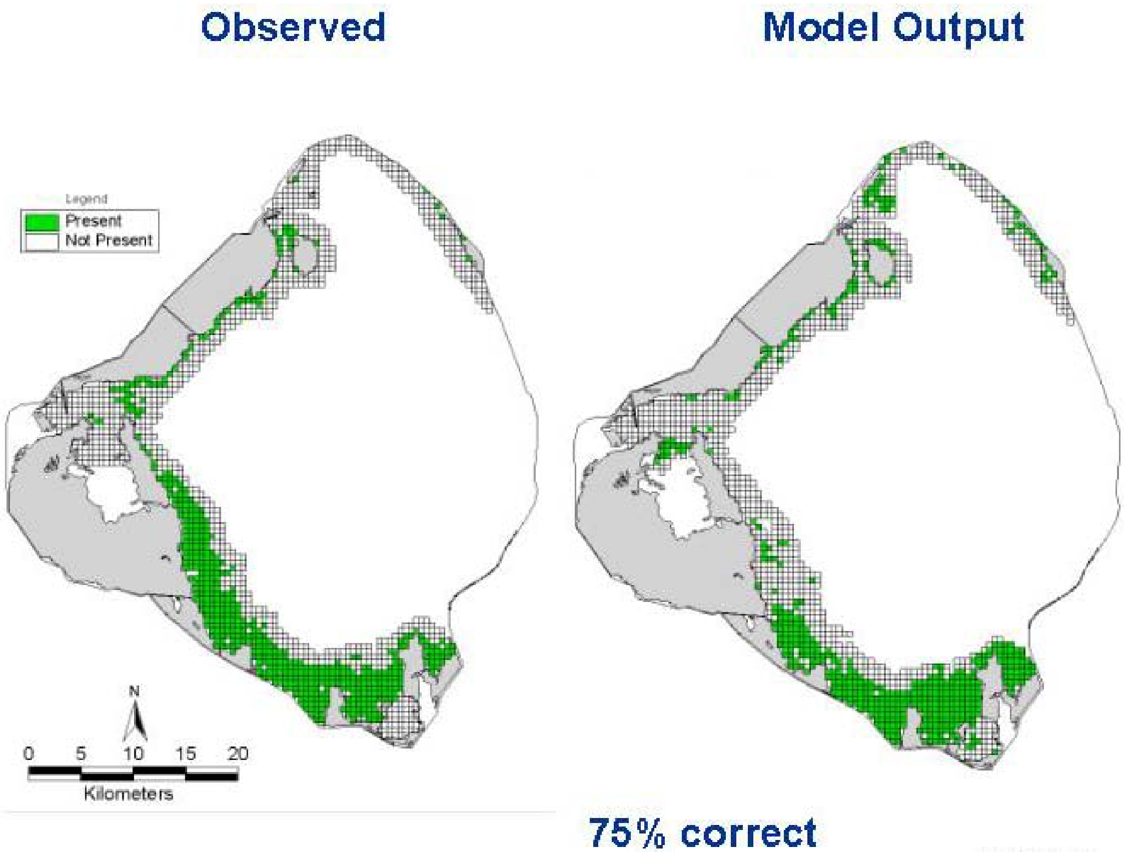

**FIGURE 4B**

Although the decision model is based on a rather simplistic calibration exercise with a one-time survey program, it accounted for 75% of the spatial variation of Chara presence/absence, and it corresponds with results from an experimental study on the ecophysiology of Chara. Steinman et al.[33] carried out photosynthesisœirradiance studies with Chara collected from the southern region of Lake Okeechobee and documented a compensation point (no net primary production) of approximately 10 μmol photons m$^{œ2}$ s$^{œ1}$. On examining recently collected data on water depth and near-bottom irradiances in the lake, we find that this corresponds to a depth of 1.5 to 2 m, the observed cutoff point for Chara occurrence in the mapping project. Similarly, Chambers and Kalff[35] developed a polynomial regression model that, when applied to Lake Okeechobee, suggested one would not expect to find Chara beyond the 1.5-m zone, given the typical Secchi depths in the near-shore region of the lake. Canfield et al.[12] developed a regression model using SAV data from lakes in Florida, Finland, and Wisconsin, and based on that model, the expected outer extent of SAV in Lake Okeechobee also is in the 1.5 to 2 m range, as observed by Steinman et al.[33].

The finding that a model based on sediment type and depth is much better in predicting Chara occurrence than is depth alone also is consistent with results of SAV research on other lakes. Ganfy and Gasith[11] evaluated SAV at a large number of sites around the perimeter of Lake Kinneret, Israel and found that the main factor controlling spatial variability was sediment type. Depth of the lake determined whether plants could attain a high biomass in any given year. In years when depth was below 1.5 to 2 m in the littoral zone, plants were able to develop a high biomass, with their location determined by the composition of sediments. Lehmann et al.[27] examined the relationships between SAV and environmental variables in Lake Geneva and found that depth and sediment type were strongly associated with the occurrence of different species of Potamogeton. In Lake Okeechobee, it is easy to discern why mud sediments did not support Chara – these sediments are highly fluid in nature and do not provide a stable substrate for plant attachment and growth. Areas with mud sediments also tended to have very poor transparency, likely caused by wind-driven resuspension of this material, which has a negligible critical shearing stress. The reason for greater Chara occurrence at any given depth in peat vs. sand is unclear and will be resolved with controlled experiments in a future study.

#### Limitations of the Sampling and Modeling Protocol

An underlying premise in the annual SAV mapping of Lake Okeechobee is that the spatial extent of plants measured in AugustœSeptember represents the yearly maximum. This same approach is used in other large-scale regional SAV survey programs, such as the Chesapeake Bay[16,17]. If the timing of the most active —growing season“ (a function of photoperiod and lake surface elevation) varies from one year to the next, this may introduce a bias into the interyear comparison. Therefore, we feel that the yearly mapping project provides information most applicable for evaluating long-term trends in SAV distribution, whereas the program of bimonthly sampling of SAV along fixed transects is better suited for identifying changes at yearly time scales. The simple decision-tree model provides meaningful results only where water depths measured at the time of SAV sampling reflect the depths that plants experienced during that summer‘s growth. In 2000, the surface elevation of Lake Okeechobee varied only slightly from mid June to mid September, an ideal situation for model development. This may not always be the case.

### CONCLUSIONS

The sampling method described here, with intensive field sampling using rapid methods coupled with automatic data logging and GIS mapping, is an effective approach for sampling SAV in turbid, shallow lakes. The presence/absence of plants can be effectively determined regardless of water clarity. An approach also was described for developing a simple decision model for predicting the occurrence of Chara across the near-shore landscape. The model accounted for 75% of the observed spatial variation, and the results were consistent with findings from studies carried out on other lakes and with ecophysiological studies of Chara in Lake Okeechobee. This decision-tree modeling approach might have general application in other lake ecosystems, but it will require validation with independent data for this and other lakes before its utility can be fully understood.

### ACKNOWLEDGEMENTS

The authors are grateful to the following persons for providing assistance in the field during the 2000 submerged vegetation mapping project: Frank Chang, Charles Hanlon, R. Thomas James, H. James Grimshaw, Kang-Ren Jin, James Laing, Kim O‘Dell, Susan Ray, and Alan Steinman. Comments from Marcel van den Berg, Peter Doering, Becky Robbins, and Alan Steinman led to improvements in an earlier version of this manuscript.
